# Demographic, economic, and social status and knowledge of medical and alternative cannabis use among Northeastern Thai citizens: a case study of outpatients at community hospitals along the Thai–Cambodian border

**DOI:** 10.1186/s42238-025-00326-3

**Published:** 2025-10-23

**Authors:** Pattarachit Choompol Gozzoli, Suyanee Pongthananikorn, Tanattha Kitisopee, Sathian Phunpon, Kamolwan Tantipiwattanasakul, Phakarat Tangkheunkan, Kannikar Khaw-ngern, Kritsada Chakchai, Niyada Kiatying-Angsulee, Shinnawat Saengungsumalee

**Affiliations:** 1 Department of Society and Health, Faculty of Social Sciences and Humanities, Mahidol University, Nakorn Prathom, Thailand; 2 Department of Food and Pharmaceutical Chemistry, Faculty of Pharmaceutical Sciences, Chulalongkorn University, Bangkok, Thailand; 3 Faculty of Pharmacy, Siam University, 38 Petchkasem Road, Bangwa, Phasicharoen, Bangkok, Thailand; 4 Graduate School, Mahachulalongkornrajavidyalaya University, Ayutthaya, Thailand; 5 Phayu Hospital, Srisaket, Thailand; 6 Drug System Monitoring and Development Centre, Social Research Institute, Chulalongkorn University, Bangkok, Thailand

**Keywords:** Cannabis use, Medical cannabis, Thailand, Knowledge

## Abstract

**Objectives:**

This study aimed to examine the demographic, economic, and social characteristics of the population in relation to their experiences with cannabis use and their accurate knowledge regarding its therapeutic properties and side effects when used in conjunction with conventional and alternative medicine and analyze the legal clarity concerning cannabis regulations and to propose communication strategies for effectively disseminating knowledge about its therapeutic properties and potential side effects when used for medical purposes.

**Method:**

A cross-sectional quantitative survey was conducted between March and August 2024 at a 30-bed community hospital along the Thai-Cambodian border. The sample included 618 Thai outpatients (429 females, 189 males) aged 18–86 years, selected via systematic structured interval-based sampling. Data were collected through face-to-face structured interviews using a validated questionnaire covering demographics, cannabis use history, and knowledge of medical uses and side effects.

**Results:**

Only 14.24% of respondents had prior cannabis use experience. While most were aware of cannabis legalization, a large proportion showed Limited understanding of its approved medical indications or side effects. Over 60% of participants answered “do not know” about therapeutic benefits or adverse effects. Differences in knowledge were found across age groups and education levels, but awareness was relatively low among health volunteers (Village Health Volunteers).

**Conclusions:**

Despite policy liberalization, knowledge gaps concerning medical cannabis remain widespread, especially regarding its safe and appropriate use. The findings highlight the need for targeted public education, regulatory clarity, and culturally appropriate communication strategies to ensure safe usage and minimize health risks, particularly in sensitive border areas.

## Introduction

The decriminalization of cannabis from a Category 5 narcotic under the Narcotic Code B.E. 2564 (2021) marked a significant turning point in Thailand’s drug policy, traditional medicine, and economic landscape. The government’s initiative aimed to increase the economic value of herbal plants, beginning with the enactment of the Narcotic Act (No. 7), B.E. 2562 (2019), which allowed cannabis to be used for medical purposes and encouraged both biomedical and socio-humanistic research for the first time (Ahmed M. etal. 2023). On June 9, 2022, the Ministry of Public Health officially removed cannabis from the list of controlled narcotics, making Thailand the first country in Asia to legalize the use of cannabis for both medicinal and recreational purposes. This legalization facilitated cannabis use in commercial, medical, and research domains without contravening prior laws (Amarin TV 2022, American Cancer Society 2023; Aroonsrimorakot et al. 2019).

Conventional medical cannabis refers to the use of cannabis-derived products, such as THC, CBD, or other cannabinoids under the supervision of licensed physicians to treat or alleviate symptoms of specific medical conditions. These include, but are not limited to, chronic pain, chemotherapy-induced nausea and vomiting, and muscle spasticity in patients with multiple sclerosis (Bank of Thailand 2023). These products are generally in the form of standardized extracts or pharmaceutical preparations and are regulated under medical cannabis programs authorized by the Ministry of Public Health. In contrast, alternative cannabis refers to the use of raw parts of the cannabis plant, such as leaves, roots, or flowers, in traditional Thai medicine formulations or alternative health products. These applications are typically integrated into Thai traditional pharmacopoeia and require approval under the Department of Thai Traditional and Alternative Medicine (Bone et al. 2024). Examples of alternative cannabis uses include treatments for treatment-resistant epilepsy, insomnia, and general tonic remedies, when used in accordance with registered traditional formulas.

This policy shift was driven mainly by increasing evidence supporting the medicinal benefits of cannabis, such as pain relief in cancer patients (Bank of Thailand 2023), treatment of epilepsy, and management of depression. It also created opportunities in industries producing dietary supplements and cosmetics. In 2021, the Department of Thai Traditional and Alternative Medicine promoted the medical use of cannabis through over 893 clinics nationwide, serving more than 7,200 patients (Bone et al. 2024). Economically, the Bank of Thailand estimated the cannabis market in 2023 to be worth approximately 28 billion THB and projected continued growth (Bottorff et al. 2013). As a result, numerous cannabis retail points have emerged nationwide. By April 2024, there were 7,747 such outlets, with Bangkok alone hosting 1,122 (Cannabis Legal News 2023). A 2023 survey revealed that 20% of the population had used cannabis, and 9% cultivated it at home. However, cannabis use among youth has rapidly increased, especially among those outside the formal education system, where usage rates reached 47.6%. This often involved concurrent use with other substances such as alcohol, tobacco, and kratom (Center for Addiction Studies 2019, Curaleaf Clinic 2023; Chong and Druckman 2007).

Although cannabis legalization was expected to yield more positive than adverse outcomes in the medical and commercial sectors, unforeseen negative consequences have emerged, particularly in public health and social domains. Hospitals have reported increasing cases of cannabis-related side effects. Public concern about cannabis misuse, especially among youth, is growing. A 2023 survey by the National Statistical Office found that 68% of the population supported stricter cannabis regulations (Thai Medical Council 2022). Consequently, there have been calls for new legislation, such as the Cannabis and Hemp Bill, to provide clearer regulatory frameworks (Cronbach et al. 1963). This aligns with findings from the Center for Addiction Studies, which identified the northeastern region as having the highest cannabis usage rates. For example, nearly two-thirds of males aged 30–59 reported using cannabis for recreational purposes, and youths aged 15–19 were found to use cannabis without restriction (de Souza et al. 2022). In this region, cannabis users also experienced mental health issues and anxiety and engaged in poly-substance use (Department of Health 2022).

The Thai-Cambodian border, part of the northeastern region, has deep historical, cultural, and economic ties, influencing local cannabis usage across various dimensions, including commerce, product mobility, and daily consumption (Department of Medical Services 2022, Department of Thai Traditional and Alternative Medicine 2021). Cannabis is commonly used as an herbal remedy and food ingredient, such as in chicken soup, spicy soups, noodles, and cookies, to relieve muscle pain through traditional recipes. However, following Thailand’s 2022 legalization, cannabis usage and trade dynamics along the border have shifted dramatically, especially in terms of product movement, as Cambodia still classifies cannabis as an illicit drug (Dominguez H. 2021). This has led to smuggling activities, and cannabis entering Thailand through unofficial channels often escapes quality control, posing consumer risks. Youths in this area have easy access, leading to improper usage that may result in long-term mental health issues and risky behaviors (Cannabis Legal News 2023; Durán-Martínez and Pennell 2024).

Therefore, while cannabis legalization has spurred the development of medical and industrial products, cultivation and production must be tightly regulated. National measures and bilateral cooperation between Thailand and Cambodia remain crucial. Moreover, the knowledge and understanding of cannabis, its benefits, risks, and side effects among local populations, from youth to older people, remain unclear. This knowledge gap is particularly significant given the fragile, risky, and potentially dangerous nature of cannabis use, especially for both users and healthcare providers in both Thailand and Cambodia. Hence, there is a pressing need to explore the demographic, economic, and social factors that influence cannabis knowledge and usage, including its applications in modern and alternative medicine and associated side effects areas that have not been systematically studied in terms of valid measurements and question formulation. This is particularly true for border regions where cannabis use predates legalization (French 2002).

Lessons from the U.S.–Mexico border illustrate how legal changes in the U.S. cannabis policy affected smuggling patterns, as legalization led to decreased demand in the black market but increased health-related side effects among users (Garcia et al. 2024; Gobbi et al. 2019; Goodman et al. 2020; Health Canada 2023). Similar patterns have been observed in Canada, where liberalization increased cannabis use across various demographics, particularly among youth with easy access (Green et al. 2017; Hall and Solowij 1998). These decisions are often influenced by misinformation, unawareness of potential side effects, misconceptions, or social trends (Headset 2018, Hfocus 2024; Hughes et al. 2001). Therefore, it is vital to investigate the demographic, economic, and social determinants of cannabis knowledge and use, especially in the context of modern and alternative medicine, among northeastern residents along the Thai-Cambodian border. Without proper knowledge assessment, unregulated cannabis use could lead to adverse physical and mental health outcomes similar to those seen in U.S. or Canadian border regions (iLaw 2022).

In Thailand, the legal framework governing the use of cannabis for medical and health-related purposes has undergone significant changes, particularly since 2019, when certain parts of the cannabis plant were removed from the narcotics schedule and permitted for use under specific conditions set by the Ministry of Public Health. Medical cannabis use must be supervised by licensed healthcare professionals within authorized medical facilities and must follow clinical guidelines issued by the Department of Medical Services. In the domain of traditional Thai medicine, specific parts of the plant can be used in approved formulations under the supervision of certified Thai traditional practitioners, by the Department of Thai Traditional and Alternative Medicine (Ahmed M. 2023, Jaeger K. 2022; Thammarangsri et al., 2023; Inkhiew et al. 2023; Jampathong et al. 2022; Johns 2001; Kamnoedrit 2022; Kichloo et al. 2021; Kronick 2020). However, ambiguities in the interpretation and enforcement of cannabis-related laws, especially in border regions, have led to inconsistent understanding and application among the public. These legal complexities motivated this study, which aims to explore the knowledge and lived experiences of community members regarding cannabis use within the local healthcare system.

This study aims to address the current knowledge gap by generating critical insights that inform accurate public education, highlight cannabis-related side effects, and support informed decision-making for therapeutic cannabis use. This is particularly important in increasing cannabis availability in border areas. Another key motivation is the limited research on cannabis use in the Thai-Cambodian border context. To date, no studies have analyzed response accuracy regarding approved medical uses of cannabis or associated side effects. Therefore, this study also aims to assess the clarity of laws related to cannabis use and propose effective communication strategies for public health messaging. These elements are essential for fostering a more accurate understanding of cannabis, ultimately guiding safe usage practices in the future.

## Method

This study adopted a quantitative cross-sectional survey design, complemented by a document-based analysis of primary legal sources issued by Thai government agencies concerning the medical and alternative medicinal use of cannabis. Data collection occurred between March 10 and August 31, 2024, during the transitional period between northeastern Thailand’s hot and rainy seasons, specifically in border districts adjacent to Cambodia. Participants were Thai outpatients aged 18–86 from a 30-bed community hospital in a border district. The study focused on this region due to its unique socio-cultural and economic with Cambodia, as well as its active cannabis usage.

Data collection was conducted on weekdays between 8.30 a.m. and 11.00 a.m. using a structured interval-based sampling method. With the permission and coordination of the hospital director and outpatient department (OPD) staff, the data collection process commenced at the weighing station, the first mandatory stop for all outpatients. The first eligible patient who passed through the weighing station and provided informed consent was selected as the index case. Subsequently, every fourth patient (k = 4) was approached for participation using the same procedure. This systematic interval-based sampling was repeated consistently to ensure a structured selection process and minimize bias. Between March 10 and August 31, 2024, the OPD served approximately 15,828 patients—averaging 140 per day. Data collection was carried out over 119 working days. Although the team initially aimed to collect data from 10 patients daily. Participants were systematically approached, approximately 20 patients per day, with a detailed oral explanation of the study objectives and ethical safeguards. Informed consent was obtained both orally and in writing. The inclusion criteria included being between 18 and 80 years old. Patients with serious illnesses, acute distress, or those unable to consent were excluded. Interviews lasted about 25–30 min. Before participation, oral consent was obtained from each potential participant during a detailed explanation of the study’s objectives, procedures, and confidentiality measures. A printed informed consent form was also prepared and offered for signature. However, most participants (*n* = 580) willingly agreed to participate in the survey without signing the form, citing a preference for avoiding the perceived formal commitment of a written signature. These participants nonetheless confirmed their voluntary participation orally. The remaining 38 participants provided both oral agreement and a signed informed consent form. This dual approach was intended to respect participant autonomy, accommodate cultural sensitivities surrounding written agreements, and ensure that participation was entirely voluntary.

Before participation, oral consent was requested from each potential participant. The researcher provided a clear explanation of the study’s purpose, procedures, and confidentiality measures. Those who expressed a willingness to participate were then invited to a designated private counseling room, located approximately three meters from the outpatient weighing station, where the hospital pharmacist was stationed. This research project was conducted with the official support of the hospital director and OPD staff to ensure the availability of appropriate facilities and procedures. Interviews were conducted one-on-one, with only the field researcher and the participant present in the room; no other hospital staff or individuals were permitted during the session, assuring privacy and reducing external influence.

Participants were also given a printed informed consent form, which they were encouraged to read independently. Signing the form was optional, and oral consent was sufficient to proceed, thereby respecting participant autonomy in a cultural context where written consent may be seen as overly formal or intimidating, especially regarding sensitive topics such as cannabis use. All respondents received both a oral explanation and a written consent form before any data collection began, and interviews were conducted only after oral consent had been granted. To safeguard participant anonymity, no names or personal identifiers were recorded; instead, a structured anonymized coding system was applied. For instance, the code P010506900F denoted the first patient interviewed (01) on June 5 (0506), beginning at 9.00 a.m. (900), and identified as female (F). This systematic approach ensured both ethical compliance and the protection of respondent confidentiality throughout the study.

### Research instrument

A structured, close-ended questionnaire was employed for data collection, administered through face-to-face interviews by a trained field researcher in private and convenient outpatient settings. The questionnaire consisted of four sections: (1) demographic information (age, sex, education, occupation, income); (2) knowledge of cannabis’s therapeutic properties in conventional and alternative medicine (Bank of Thailand 2023; Kroon et al. 2020; Langka W. 2024), measured using three response options (correct, incorrect, don’t know); (3) knowledge of cannabis-related side effects (Laranjeira and Martin 2019; Li et al. 2023; López López et al. 2016; Mead 2017), also measured using the same three response categories; and (4) cannabis use experiences, covering medical, recreational, culinary, and commercial purposes. For analysis, the use experience was dichotomized into “ever used” and “never used” to better assess trends following legalization in June 2022. Content validity was reviewed by four experts, two in pharmaceutical sciences, one in medicine, and one in behavioral science, yielding an Index of Congruence (IOC) of 0.9. Reliability analysis of the questionnaire yielded strong internal consistency across its knowledge components. Overall, the knowledge-related items produced a Cronbach’s alpha of 0.83, reflecting acceptable reliability (approximately 69%) (Ministry of Public Health 2021). To further assess measurement robustness, the analysis was conducted separately for two key domains: items related to knowledge of the therapeutic effects of cannabis produced a Cronbach’s alpha of 0.89, and items addressing adverse events yielded a Cronbach’s alpha of 0.88.

### Data analysis

Descriptive statistics were used to summarize the characteristics of the study population. Categorical variables, including gender, age group, education level, and occupation, were described using frequencies and percentages. Additional variables, such as awareness of medical cannabis and reported side effects, were also summarized in this manner. To explore associations between independent variables and key cannabis-related outcomes, particularly lifetime experience with medical cannabis use, the Chi-square test (χ^2^) was employed. A two-tailed p-value of less than 0.05 was considered statistically significant. Bivariate analyses were initially conducted using Chi-square tests to identify significant associations between categorical independent variables and the dependent variable.

Nevertheless, the chi-square test has certain limitations, as it is essentially a one-by-one analysis that examines the relationship between one independent variable and the dependent variable without simultaneously accounting for the influence of other variables. To address this limitation, a logistic regression analysis was employed as a multivariate approach to explore the combined effects of multiple factors on the experience of cannabis use.

The logistic regression analysis was conducted using the Enter method, which allowed all variables to be simultaneously included in the model. This approach was designed to evaluate the overall contribution of each variable to predicting prior cannabis use. Importantly, variables were retained in the model even if they did not reach statistical significance, provided that their inclusion contributed to a significant increase in the model’s Chi-square statistic. This procedure ensured that the selected independent variables were adequately represented, while also confirming statistical independence and the absence of problematic collinearity or multicollinearity.

To further account for potential confounding factors and to determine the adjusted effects of each predictor, a multivariate logistic regression analysis was performed. The dependent variable was defined as cannabis use experience (coded as 1 for ever used, and 0 for never used). Independent variables entered into the model included gender (Male = 0, Female = 1), age group (18–39 years = 1, others = 0; 40–54 years = 1, others = 0; with ≥ 55 years as the reference group), education level (Below basic = 1, others = 0 as the reference group; Basic = 1, others = 0; Above basic = 1, others = 0), and occupation (Village Health Volunteers (VHVs) = 1, others = 0). Odds ratios (ORs) with corresponding 95% confidence intervals (CIs) were calculated to interpret the direction and strength of associations. All statistical analyses were conducted using IBM SPSS Statistics version 29.0.1.0 (Build 171), with a significance level of *p* < 0.05 (two-tailed).

## Results

### 1) Sample characteristics

The structured interval-based sampling method yielded an average of five respondents per day, resulting in a total of 618 completed responses over the data collection period. Based on the daily number of patients approached and actual participation, the estimated refusal rate was approximately 25% per day and 28% overall.

In contrast, those who declined participation were primarily male, aged 20–39 years, and employed as wage laborers or government officials, many of whom appeared to have higher educational attainment. This was inferred from their uniforms and medical benefits (e.g., civil servant medical benefit scheme). These characteristics suggest a potential participation bias influenced by occupation, gender, and perceived stigma or time constraints surrounding cannabis-related topics.

Among the 618 participants, an apparent gender disparity was observed: 429 respondents (69.4%) were female, while only 189 (30.6%) were male. This imbalance reflects a broader societal trend in which women are generally more proactive in health-seeking behaviors and outpatient service engagement. Age groups were categorized into three generational cohorts for comparative analysis: youth and young adults (ages 18–39, Generation Y and Z) accounted for 73 participants; adults (ages 40–54, Generation X) represented the majority with 457 participants; and older adults (ages 55–77, Baby Boomers) comprised 88 participants. This classification facilitates an exploration of generational differences in legal consciousness, social norms, and ideological tendencies, liberal versus conservative, shaped by distinct sociolegal environments during formative years. Occupationally, the sample was skewed toward village health volunteers (VHVs), who play a significant role in Thailand’s public health infrastructure, particularly in disease surveillance and community-based health promotion. Other professions, such as farmers and daily laborers, were represented in smaller proportions.

Regarding education, respondents who had completed at least compulsory education were more likely to demonstrate cognitive competencies, including legal awareness, literacy in foreign languages (especially English), and analytical reasoning. Cannabis use experience categorized as medical, recreational, culinary, or commercial was considered a proxy for practical knowledge and understanding of cannabis’s benefits and side effects. As detailed in subsequent sections, these experiences were further analyzed across demographic, economic, and social variables.

### 2) Demographic, cconomic, and social characteristics and cannabis use experience

Cannabis use experience was considered a critical behavioral indicator in this study, reflecting not only actual consumption but also its potential correlation with knowledge accuracy regarding therapeutic benefits and side effects. Before its decriminalization in 2021, cannabis use in Thailand was a criminal offense punishable by up to five years imprisonment or a fine of 500,000 THB (Ahmed M. 2023).

Analysis of usage patterns alongside demographic, economic, and social variables revealed a significant disparity, with non-users outnumbering users nearly five to one among female and male respondents. Generation X (ages 40–54) reported the highest usage rates compared to other cohorts. Yet, overall cannabis use remained low, especially among village health volunteers (VHVs), who are closely affiliated with state health services and likely cautious due to lingering legal ambiguity. Education level also influenced behavior, with those having more than basic education reporting the lowest usage. Even among participants with primary education or less, cannabis use remained minimal.

These trends suggest that historical legal restrictions and sustained confidence in conventional medicine continue to shape public attitudes. As detailed in Table 1, most respondents were women over 40, working in community health roles with Limited formal education, and only 88 individuals (14.24%), approximately one in seven, reported any prior cannabis use. When considering gender differences in cannabis use and non-use, one of the most notable findings from this study is that being male or female significantly influences such behavior and is statistically associated with cannabis use experience. Specifically, among those who had never used cannabis, the number of female participants was higher than that of males. Interestingly, even among those who had used cannabis, the proportion of females was also higher than that of males. The chi-square test showed a statistically significant relationship between gender and cannabis use experience (χ^2^ = 10.7, df = 1, *p* < 0.05). However, it is important to note that differences in age group (χ^2^ = 3.8, df = 2), education level (χ^2^ = 1.7, df = 1), and occupation group (χ^2^ = 0.2, df = 1) were not statistically significant regarding cannabis use. However, this finding may be regarded as a result derived solely from the bivariate analysis, which does not yet account for the combined influence of other factors through a multivariate analysis.

**Table 1:** Sociodemographic characteristics and cannabis use experience

Sociodemographic characteristics	Distribution characteristics		Cannabis use experience		
	**Number (***n* **= 618)**	**Percentage (%)**	**Never used (***n* **= 530)**	**Ever used (***n* **= 88)**	χ^2^** (D.F.)**
**Gender**					10.7^*^ (1)
Male	189	30.6	149 (78.8)	40 (21.2)	
Female	429	69.4	381 (88.8)	48 (11.2)	
**Age**					3.8 (2)
18–39 year old	73	11.8	58 (79.5)	15 (20.5)	
40–54 year old	457	73.9	399 (87.3)	58 (12.7)	
55-year-old above	88	14.2	73 (83.0)	15 (17.0)	
**Occupation**					0.2 (1)
Village Health Volunteers (VHVs)	377	61.0	355 (86.2)	52 (13.8)	
Others (e.g., farmers, daily wage workers)	241	39.0	205 (85.1)	36 (14.9)	
**Education**					1.7 (1)
Below basic education level	223	36.1	190 (85.2)	33 (14.8)	
Basic education level	362	58.6	314 (86.7)	48 (13.3)	
Above basic education level	33	5.3	26 (78.8)	7 (21.2)	

^*^ Statistically significant

Based on the results of the Chi-square test presented above, which was conducted as part of the bivariate analysis, only gender was found to have a statistically significant association with prior cannabis use. In contrast, age group, occupation, and educational attainment did not show any significant associations with the dependent variable. Therefore, a multivariate analysis was subsequently performed using logistic regression, as the dependent variable was measured as a binary outcome (1 = ever used, 0 = never used). The independent variables were measured according to the definitions and procedures described in the methodology section. It is important to note that multivariate analysis requires certain assumptions: each independent variable must demonstrate a relationship with the dependent variable, and, critically, the independent variables must be statistically independent of each other, without evidence of collinearity or multicollinearity. To address these assumptions, Pearson’s correlation analysis was conducted, with a cut-off point set at 0.650. The results of this test are presented in Table 2.

**Table 2:** Pearson correlation matrix of demographic variables (*N* = 618)

Variables	1	2	3	4	5	6	7	8	9
1. Cannabis use experience	1								
2. Gender	−1.320**	1							
3. VHVs	−0.016	−0.019	1						
4. 18–39 year old	0.066	0.047	−0.016	1					
5. 40–54 year old	−0.40	0.123**	−0.010	−0.394**	1				
6. 55-year-old above	−0.003	−0.162**	.021	−0.265**	−0.781**	1			
7. Below basic education level	0.012	−0.035	−0.097*	−0.191**	−0.086*	0.221**	1		
8. Basic education level	−0.033	0.048	0.068	0.074	0.122**	−0.178**	−0.893**	1	
9. Above basic education level	0.047	−0.030	0.057	0.248**	−0.083*	−0.081*	−0.178**	−0.282**	1

^*^p < 0.05, **p < 0.01 (2-tailed), *N* = 618

Table 2 presents the Pearson correlation matrix among demographic variables included as independent predictors in the logistic regression model, which analyzes factors associated with prior cannabis use among 618 participants. Before model estimation, collinearity and multicollinearity were assessed using a cut-point threshold of 0.650. Results revealed a strong negative correlation between the variable “40–54-year-old” and the reference group “55-year-old above” (r =—0.781), as well as between “Basic education level” and “Below basic education level” (r =—0.893), as shown in Table 2. To mitigate the risk of multicollinearity, the variable “55-year-old above” was used as the reference category for age, and “Below basic education level” was used as the reference category for education. Interestingly, when considering the Pearson correlation between gender (female = 1, male = 0) and prior cannabis use, a highly notable finding emerged: the correlation was negative (r = −1.320, *p* < 0.001), indicating that being female was negatively associated with the experience of ever using cannabis. In contrast, other factors such as occupation as a village health volunteer, age group, and educational level did not show statistically significant associations with cannabis use. It is also worth noting that the observed correlation coefficients were relatively low but not zero, suggesting that these independent variables may have some relationship with cannabis use, albeit weak.

Table 3 presents the results of a multivariate logistic regression analysis, which revealed that gender, after controlling for age group, occupation, and educational level, was the only statistically significant predictor of prior cannabis use. Females were significantly less likely to have used cannabis compared to males (B = −0.779, p = 0.001, OR = 0.459, 95% CI [0.287, 0.735]).

**Table 3:** Logistic regression predicting outcome variable (*N* = 618)

Predictor	B	SE	Wald	df	*p*-value	OR (Exp(B))	95% CI for OR
Female	−0.779	0.240	10.552	1	0.001***	0.459	[0.287, 0.735]
Age 18–39	0.590	0.384	2.363	1	0.124	1.804	[0.853, 3.812]
Age 40–54	0.058	0.267	0.046	1	0.829	1.059	[0.632, 1.774]
VHVs	−0.088	0.240	0.133	1	0.716	0.916	[0.576, 1.459]
Basic education	−0.155	0.256	0.366	1	0.545	0.856	[0.517, 1.416]
Above basic education	0.183	0.506	0.132	1	0.717	1.201	[0.445, 3.240]
Constant	−1.282	0.288	19.780	1	< 0.001***	0.277	-

−2 Log likelihood = 491.808, Model Chi-square = 14.070, DF = 6, Sig. = 0.029, Nagelkerke R Square = 0.040, *** *p* < 0.001

From Table 3 although the variable age 18–39 years did not reach statistical significance (*p* = 0.124), the odds ratio (OR = 1.804) and its 95% confidence interval [0.853, 3.812] suggest a noteworthy trend. Individuals in this younger age group were nearly twice as likely to have used cannabis compared to those aged ≥ 55 years. This trend may reflect generational differences in attitudes toward cannabis use, increased exposure to pro-cannabis messaging through social media, and recent legal reforms in Thailand that have made cannabis more accessible. Curiosity and experimentation, particularly among youth and early working-age adults, may also explain this pattern.

Other variables, such as being a village health volunteer (VHV) and education level, were not statistically significant but were retained in the model under the enter method approach. This method emphasizes the overall predictive power of all included variables rather than limiting the model based solely on statistical significance. If a variable contributes to increasing the model’s Chi-square statistic, it may hold practical predictive value even in the absence of significance.

### 3) Demographic, economic, and social characteristics and knowledge of cannabis's therapeutic properties in conventional medicine

Although only 88 of 618 participants (14.24%) reported prior cannabis use, their responses revealed that direct experience did not strongly correlate with accurate knowledge of cannabis’s therapeutic applications. This group’s most common uses were muscle inflammation and sleep aids, while recreational, culinary, and commercial uses were less frequent. Despite Thailand’s legalization of cannabis in June 2022 (Ministry of Public Health 2021), knowledge of its medical indications, such as treating chemotherapy-induced nausea, improving quality of life in terminal illness, and alleviating muscle spasticity, remained limited, with correct responses comprising only about one-sixth of the sample and “don’t know” being the dominant answer across all items. Interestingly, cannabis use history, gender, education level, or occupation did not significantly improve accuracy.

However, older respondents (Generation X and Baby Boomers) showed slightly better knowledge, potentially due to accumulated experience or caregiving roles. Uncertainty prevailed even among village health volunteers (VHVs), who are typically expected to possess greater health literacy. These findings highlight a widespread knowledge gap across all demographic sectors and underscore the urgent need for structured, evidence-based public education strategies to support informed decision-making regarding medical cannabis use. Table 4 presents sociodemographic characteristics and knowledge of cannabis’s therapeutic properties in conventional medicine.

**Table 4:** Demographic, economic, and social characteristics and knowledge of cannabis’s therapeutic properties in conventional medicine

Sociodemographic characteristics	Cannabis use for alleviating chemotherapy-induced nausea and vomiting			Cannabis use for improving the quality of life in terminally ill patients			Cannabis use for treating muscle spasticity in patients with multiple sclerosis		
	**Incorrect**	**Correct**	**Don’t know**	**Incorrect**	**Correct**	**Don’t know**	**Incorrect**	**Correct**	**Don’t know**
**Gender**									
Male	20 (10.6)	50 (26.5)	119 (63.0)	29 (15.3)	53 (28.0)	107 (56.6)	17 (9.0)	48 (25.4)	124 (65.6)
Female	39 (9.1)	109 (25.4)	281 (65.5)	53 (12.4)	128 (29.8)	248 (57.8)	45 (10.5)	100 (23.3)	284 (66.2)
**Age**									
18–39 year old	9 (12.3)	27 (37.0)	37 (57.5)	6 (8.2)	37 (50.7)	30 (41.1)	5 (6.8)	25 (34.2)	43 (58.9)
40–54 year old	45 (9.8)	112 (24.5)	300 (65.6)	60 (13.1)	127 (27.8)	270 (59.1)	47 (10.3)	101 (22.1)	309 (67.6)
55-year-old above	5 (5.7)	20 (22.7)	63 (71.6)	16 (18.2)	17 (19.3)	55 (62.5)	10 (11.4)	22 (25.0)	56 (63.6)
**Occupation**									
Village Health Volunteers (VHVs)	35 (9.3)	103 (27.3)	239 (63.4)	49 (13.0)	117 (31.0)	211 (56.0)	41 (10.9)	94 (24.9)	242 (64.2)
Others (e.g., farmers, daily wage workers)	24 (10.0)	56 (23.2)	161 (66.8)	33 (13.7)	64 (26.6)	144 (59.8)	21 (8.7)	54 (22.4)	166 (68.9)
**Education**									
Below basic education level	26 (11.7)	43 (19.3)	154 (69.1)	36 (16.1)	49 (22.0)	139 (61.9)	28 (12.6)	39 (17.5)	156 (70.0)
Basic education level	30 (8.3)	100 (27.6)	232 (64.1)	44 (12.2)	114 (31.5)	204 (56.4)	32 (8.8)	97 (26.8)	233 (64.4)
Above basic education level	3 (9.1)	16 (48.5)	14 (42.4)	2 (6.1)	18 (54.5)	13 (39.4)	2 (6.1)	12 (36.4)	19 (57.6)
**Cannabis use experience**									
Ever used	50 (9.4)	126 (23.8)	354 (66.8)	73 (13.8)	144 (27.2)	313 (59.1)	51 (9.6)	123 (23.2)	356 (67.2)
Never used	9 (10.2)	33 (37.5)	46 (52.3)	9 (10.2)	37 (42.0)	42 (47.7)	11 (12.5)	25 (28.4)	52 (59.1)

### 4) Demographic, economic, and social characteristics and cannabis use experience concerning knowledge of cannabis’s therapeutic properties in alternative medicine

This section explores participants’ knowledge of cannabis’s therapeutic properties in alternative medicine, including traditional Thai and local healing practices. Although cannabis has historically been used to treat conditions such as drug-resistant epilepsy in children and chronic insomnia in adults, this study found that accurate knowledge of such uses remains limited across all demographics. A significant number of participants responded with “don’t know,” a trend consistent across age, occupation, education level, and cannabis use history. Notably, prior cannabis use did not significantly correlate with correct responses regarding these therapeutic applications, suggesting that residual stigma and legal ambiguity continue to hinder public understanding. Older participants, who might be expected to demonstrate greater insight due to life experience or caregiving roles, showed similarly low knowledge levels, implying that entrenched legal and cultural taboos remain influential.

Although village health volunteers (VHVs) expected to have higher exposure to medical education performed slightly better than other occupational groups, uncertainty still dominated their responses. This pattern underscores a broader failure of both formal and informal communication systems, such as public announcements, community outreach, and local government messaging, to deliver clear, evidence-based information about cannabis in alternative medicine.

Ongoing regulatory uncertainty and the absence of national policy integration limit the diffusion of accurate knowledge. Table 5 presents a detailed breakdown of participant responses on cannabis’s role in managing epilepsy and insomnia, revealing persistent knowledge gaps across demographic and occupational segments.

**Table 5:** Demographic, economic, and social characteristics and cannabis use experience concerning knowledge of cannabis’s therapeutic properties in alternative medicine

Sociodemographic characteristics	Treatment-resistant epilepsy			Insomnia in adults		
	**Incorrect**	**Correct**	**Don’t know**	**Incorrect**	**Correct**	**Don’t know**
**Gender**						
Male	40 (21.2)	28 (14.8)	121 (64.0)	25 (13.2)	70 (37.0)	94 (49.7)
Female	72 (16.8)	71 (16.6)	286 (66.7)	55 (12.8)	138 (32.2)	236 (55.0)
**Age**						
18–39 year old	16 (21.9)	17 (23.3)	40 (54.8)	11 (15.1)	33 (45.2)	29 (39.7)
40–54 year old	81 (17.7)	66 (14.4)	310 (67.8)	58 (12.7)	148 (32.4)	251 (54.9)
55-year-old above	15 (17.0)	16 (18.2)	57 (64.8)	11 (12.5)	27 (30.7)	50 (56.8)
**Occupation**						
Village Health Volunteers (VHVs)	81 (21.5)	60 (15.9)	236 (62.6)	51 (13.5)	147 (39.0)	179 (47.5)
Others (e.g., farmers, daily wage workers)	31 (12.9)	39 (16.2)	171 (71.0)	29 (12.0)	61 (25.3)	151 (62.7)
**Education**						
Below basic education level	41 (18.4)	28 (12.6)	154 (69.1)	37 (16.6)	60 (26.9)	126 (56.5)
Basic education level	67 (18.5)	58 (16.0)	237 (65.5)	41 (11.3)	125 (34.5)	196 (54.1)
Above basic education level	4 (12.1)	13 (39.4)	16 (48.5)	2 (6.1)	23 (69.7)	8 (24.2)
**Cannabis use experience**						
Ever used	91 (17.2)	81 (15.3)	358 (67.5)	70 (13.2)	170 (32.1)	290 (54.7)
Never used	21 (23.9)	18 (20.5)	49 (55.7)	10 (11.4)	38 (43.2)	40 (45.5)

### 5) Knowledge of cannabis’s therapeutic properties and side effects when used in conventional medical treatmentand alternative medicine

Understanding both the therapeutic benefits and side effects of cannabis when used alongside conventional medicine and alternative medicine is essential, particularly for healthcare providers, patients, caregivers, and village health volunteers (VHVs), who often gain firsthand insights into patient outcomes through follow-up visits and clinical interactions. Therefore, it is expected that those engaged in patient care would possess more accurate knowledge of cannabis’s therapeutic applications and potential risks. However, this study revealed significant knowledge gaps in all areas assessed, including three medical cannabis applications endorsed by Thailand’s Department of Medical Services: managing chemotherapy-induced nausea, improving quality of life in terminal illness, and alleviating muscle spasticity in multiple sclerosis, and four common side effects: constipation, dizziness and nausea, hallucinations, and dry mouth (Laranjeira and Martin 2019; Li et al. 2023; López López et al. 2016; Mead 2017).

The data show that most of VHVs suggesting a broader deficit in public cannabis health literacy. Information from Table 6 reveal that only 159 respondents correctly identified cannabis’s role in treating chemotherapy-induced nausea, and just 181 and 148 respondents answered correctly regarding its effectiveness in improving end-of-life care and relieving muscle spasticity, respectively. Likewise, knowledge of side effects was similarly Limited; in the case of chemotherapy-induced nausea, where constipation is a known adverse effect, only 87 participants answered correctly.

**Table 6:** Knowledge of cannabis’s therapeutic properties and side effects when used in conventional medical treatment

Knowledge of therapeutic uses	Number of respondents able to answer correct knowledge of adverse effects				Total (*N* = 618)
	**Constipation**	**Dizziness and nausea**	**Hallucinations**	**Dry mouth**	
**Conventional medical treatment**					
** Alleviating chemotherapy-induced nausea and vomiting**	87 (54.7)	126 (79.2)	128 (80.5)	127 (79.9)	**159**
** Improving the quality of life in terminally ill patients**	71 (39.2)	128 (70.7)	129 (71.3)	127 (70.2)	**181**
** Treating muscle spasticity in patients with multiple sclerosis**	79 (53.4)	114 (77.0)	125 (84.5)	121 (81.8)	**148**
**Alternative medicine treatment**					
** Treatment-resistant epilepsy**	58 (58.6)	81 (81.8)	81 (81.8)	86 (86.9)	**99**
** Insomnia in adults**	84 (40.4)	158 (76.0)	163 (78.4)	156 (75.0)	**208**

Similar uncertainty was observed regarding dizziness, hallucinations, and dry mouth. Notably, those with correct knowledge of therapeutic applications were also more likely to recognize associated side effects, highlighting the influence of experiential learning. These findings underscore the compounded impact of legal ambiguity, fragmented regulatory messaging, and insufficient health communication, all of which hinder the development of an accurate, community-level understanding of cannabis in medical contexts.

As summarized in Table 6, this limited awareness highlights the urgent need for targeted, evidence-based educational strategies and clearer public policy to support informed and safe cannabis use, particularly in border regions where access to formal health information may be constrained.

Considerate on knowledge of cannabis’s therapeutic properties in alternative medicine and associated side effects, the findings from Table 6 above present that public knowledge regarding the side effects of cannabis use, particularly in the context of alternative medicine such as traditional Thai and folk healing, remains limited. Despite cannabis’s longstanding role in community-based practices informed by local wisdom, few participants in this study demonstrated accurate knowledge of its therapeutic use for conditions Like treatment-resistant epilepsy in children or severe seizures. For example, only 112 participants correctly identified these applications, underscoring that only one in six held accurate information despite increasing national discourse on medical cannabis. This knowledge gap is likely the result of insufficient public communication about cannabis’s therapeutic potential for complex neurological conditions. A similar pattern was observed regarding insomnia, with only 208 participants (about one-third) answering correctly, suggesting that informational outreach remains ineffective.

Likewise, information in Table 6 presents knowledge of cannabis’s therapeutic properties in alternative medicine and associated side effects. The finding confirms that there are not many respondents able to answer correctly, highlighting a lack of exposure to validated, evidence-based knowledge. Awareness of side effects was equally poor; few participants recognized common adverse reactions such as constipation, dizziness, nausea, hallucinations, and dry mouth. These results underscore a widespread lack of understanding regarding both the therapeutic and physiological impacts of cannabis. Particularly concerning are neurological side effects, which affect the central nervous system, and autonomic side effects, such as dry mouth and constipation, which can pose risks if not adequately understood. Promoting cannabis’s integration into alternative medicine thus requires not only legal clarity but also transparent, culturally relevant, and medically accurate public education to ensure safe and informed use.

### 6) Communication strategies for disseminating knowledge on therapeutic properties and side effects

Effective communication is essential to promote safe and informed cannabis use, particularly in border regions such as those between Thailand and Cambodia. To this end, the study proposes utilizing the SMCR model (Source–Message–Channel–Receiver) to guide the design and delivery of public health information on medical cannabis. The findings suggest that knowledge gaps are especially prominent among individuals with limited education or no prior cannabis use, making tailored communication strategies necessary.

In the Source component, trusted figures from policy-level organizations, such as senior executives from the Ministry of Public Health, Ministry of Justice, Ministry of Interior, and Ministry of Agriculture, should serve as primary messengers. These figures should be supported by community-level medical professionals such as doctors and pharmacists. Equally important are local authority figures, provincial governors, district chiefs, village leaders, and spiritual leaders, including traditional healers and religious figures. These individuals should be trained to convey both the therapeutic potential and risks of cannabis based on scientific evidence, such as the National Academies of Sciences (Ministry of Public Health 2021) report, which recognizes cannabis’s effectiveness in alleviating chemotherapy-induced nausea and chronic pain.

In the Message dimension, content must present a balanced perspective on benefits and risks. While some participants were aware of cannabis’s usefulness in treating specific conditions such as chronic pain or epilepsy, few understood the potential side effects of dizziness, nausea, hallucinations, dry mouth, and constipation. Therefore, communication materials, print, digital media, and infographics should integrate dual-framing techniques (Ministry of Public Health 2022), presenting positive and negative aspects to support informed decision-making. Messages should be phrased in simple language, accompanied by visual aids suitable for audiences with basic literacy levels.

In terms of Channel, person-centered communication remains paramount. Policy leaders and community figures should disseminate information directly, supplemented by institutional platforms such as hospital Facebook pages, community LINE groups, local radio stations, village meetings, and public address systems. These diverse formats ensure broad reach, particularly in rural areas with limited digital infrastructure.

Finally, the Receiver refers to the target audience of adults aged 18 to 77 with basic literacy and no hearing impairments, including many VHVs. Since most respondents reported limited prior cannabis use and little knowledge of its side effects, communication strategies must consider their socio-cultural and educational background. Thus, knowledge dissemination must focus on message content and give equal attention to the credibility and accessibility of messengers and communication channels.

## Discussion

This study revealed that most respondents were female village health volunteers (VHVs) aged 40 and older, representing the Generation X cohort, who have lived through Thailand’s digital and technological transformation. Despite being part of a generation that is highly integrated into the digital world, often described as digitally literate and attuned to the Internet of Things and artificial intelligence, these individuals reported limited experience with cannabis, whether for medical, recreational, or culinary purposes. This finding suggests that even within a technologically connected population, trust in conventional medicine remains dominant, mainly due to the widespread perception that modern medical technologies are more reliable and safe (Ministry of Public Health 2021, Ministry of Public Health 2022, Ministry of Public Health 2022). The prevailing public health message, “Do not self-medicate consult a physician or pharmacist,” reinforces this perspective and discourages unsupervised cannabis use (Mize M. 2020, Nantaratipob N. 2022; Morris et al. 2014; National Academies of Sciences, Engineering, and Medicine 2017).

However, when considering the findings of this study, it is particularly noteworthy that the association between gender (female = 1, male = 0) and the experience of ever using cannabis, as demonstrated through multivariate analysis, was statistically significant and negative (B = −0.779, p = 0.001, OR = 0.459, 95% CI [0.287, 0.735]). This indicates that females are less likely to have ever used cannabis compared to males. Such a finding is of great interest, as previous research has suggested that women are generally more concerned with maintaining their social image (Nelson 2021; Nukulkij 2020). Moreover, women often hold central roles in family care; for instance, married women typically carry greater responsibilities for child-rearing compared to men. Consequently, recreational cannabis use among women may be subject to greater criticism and stigmatization, being perceived as drug misuse, unless it is prescribed for medical purposes under the supervision of modern or alternative medicine practitioners (National Statistical Office 2023; Orjuela-Rojas et al. 2021).

Another explanation for this phenomenon may lie in the Thai cultural context, where the prevalence of substance use is consistently lower among women than men. This is largely due to men’s greater tendency toward risk behaviors. In Thai society, men are more likely to engage in social drinking, experimentation with new substances, and recreational cannabis use. Additionally, men often have greater social network access to peers who facilitate entry points to cannabis availability, thereby making cannabis more accessible to them than to women.

Moreover, it is particularly noteworthy that the findings of this study indicate persistent limitations in knowledge regarding cannabis, especially in terms of its therapeutic efficacy and side effects, among populations residing in the Thai–Cambodian border area. For example, the data also showed that relatively few respondents could accurately identify cannabis’s therapeutic benefits or its side effects when used alongside conventional or alternative medicine. This limited knowledge reflects a lack of engagement with cannabis-related content, likely resulting from longstanding social and legal stigmas. Similar patterns have been reported in countries such as Colombia (Pasha et al. 2021; Pearshouse and Klein 2021; Ritmontree S. 2024), Uruguay (Rodríguez-Llach et al. 2021), Brazil (Rotermann 2020; Royal Thai Government 2019), and Venezuela (Ruiz 2010), where public confusion over cannabis-related laws has contributed to poor health literacy and low utilization of medical cannabis (Samorapoom et al. 2023; Sideli et al. 2021).

In the Thai context, the public remains uncertain about what constitutes legal or illegal cannabis use. Kamnoedrit emphasized the need for legal clarity under Thai sovereignty to enable the integration of cannabis products into both conventional and alternative medical practices (Sornpaisarn et al. 2023). Similarly, Sornpaisarn et al. found that overlapping and complex legal texts have led to widespread confusion, discouraging people from learning about or using cannabis safely (Suphanchaimat et al. 2023). Samorapoom, Pewchan, & Promrit added that ambiguous legal language has created distrust, causing the public to avoid the topic entirely (Suranatwatchawong et al. 2023).

This confusion extends to the legal status of cross-border cannabis transport. Many are unaware of which countries permit or prohibit cannabis imports and exports, and this lack of clarity impacts willingness to learn about its therapeutic uses (MNI Targeted Media Inc. 2020, Thailand Development Research Institute 2024, Thairath 2024). Public hesitancy may also stem from fears that cannabis policy is politically contested and unstable or subject to change (Ministry of Public Health 2022, U.S. Food and Drug Administration 2023, Unhavaithaya J., Ditthapan S. 2023). For example, some countries such as Japan and the United Kingdom strictly prohibit cannabis importation, while in the United States, it is allowed only in specific states (UNODC 2023; Urada et al. 2020; Whiting et al. 2015).

These findings suggest that legal uncertainty, both domestic and international, significantly undermines public interest in cannabis’s medical potential and contributes to inconsistent knowledge about its uses and effects. For residents in Thailand’ northeastern border regions, special ambiguity and undefined terms related to both medical and recreational cannabis have had a measurable impact on how individuals engage with cannabis-related information.

As such, the study recommends that communication strategies be developed using the SMCR model (Source–Message–Channel–Receiver), emphasizing the need for clear legal frameworks and the dissemination of accurate, accessible information. The Thai government, particularly the Ministers of Public Health and the Ministers of Justice, should publicly define legal cannabis usage and publish an official list of countries where cross-border cannabis transport is permitted. Educational guides should also be created to improve public interest, knowledge, and safe usage of cannabis.

Additionally, influential figures such as provincial governors, district officers, village heads, traditional healers, and religious leaders should play a central role in providing centrality-based information to the public. Their involvement will not only enhance community trust but also ensure that cannabis education reaches rural populations who are most affected by inconsistent laws and limited access to health information.

### Implications and future directions

The implications of this study are twofold: first, it calls for the Thai government to urgently address the legal and informational gaps that hinder public understanding and safe use of medical cannabis, particularly in rural and border communities where misinformation thrives. Legal reforms should prioritize harmonizing terminology, clarifying permissible uses, and publishing user-friendly guidelines on what is allowed, ambiguous, or prohibited—especially regarding cross-border transport. Second, future research should expand to qualitative investigations that explore how cultural, spiritual, and generational factors influence cannabis perceptions and behaviors, particularly among youth and local healers. Educational interventions should be developed using the SMCR communication model to ensure that messages are contextually relevant, delivered by trusted community figures, and sensitive to literacy levels. A longitudinal follow-up study could assess whether such interventions improve cannabis health literacy and lead to more equitable and evidence-based use in both modern and traditional healthcare systems.

### Strengths

1. Data were collected from outpatients at a community hospital located along the Thai Cambodian’s border, an area where cannabis use spans both medical applications rooted in local traditional practices and recreational purposes, including cross-border influences. The shared cultural practices and informal movement of cannabis across the border contribute to the unique relevance and richness of the data obtained.
2. Interviews were conducted in private counseling rooms, ensuring a high level of confidentiality. Participants were clearly informed that their identities would remain anonymous using coded identifiers, which helped build trust and likely improved the accuracy and honesty of responses, particularly given the sensitivity of the topic.
3. The use of structured interval-based sampling—by inviting every fourth patient following the weight-check station—helped to minimize selection bias. This method was based on the natural flow of patients through a standard clinical process, ensuring that selection was not influenced by the researcher’s preference. It is important to note that not every fourth patient agreed to participate, which adds a degree of randomness within the systematic approach.

### Limitations

1. The sample included a disproportionate number of female participants, many of whom were village health volunteers (VHVs) with relatively low levels of formal education. This demographic distribution may reflect the actual population characteristics of healthcare service users in the area, but it also introduces a potential sampling bias.
2. The findings may be skewed toward the views and experiences of women and laypersons, while potentially underrepresenting perspectives of men, civil servants, and individuals with higher educational attainment. These limitations suggest that the generalizability of the results may be restricted to populations with similar sociodemographic profiles.
3. The relatively low participation rate, with only approximately one-quarter of eligible patients taking part, may have introduced selection bias and reduced the representativeness of the findings. Observable differences between participants and non-participants were noted; participants appeared more likely to be female, middle-aged or older, and VHVs, whereas non-participants seemed more often male, younger adults, and wage laborers or government officials. However, it should be emphasized that these impressions were based solely on informal observations and casual conversations during data collection, rather than on systematically collected data. As such, they should be interpreted with caution, and future studies should incorporate more rigorous strategies to assess differences between participants and non-participants.

## Conclusion

The empirical findings of this study indicate that females are less likely to have ever used cannabis compared to males, even after controlling for other socioeconomic factors such as age group, education level, and occupation. This result reflects broader social norms and the cultural context in Thailand. Moreover, the study underscores the persistent and widespread lack of accurate knowledge regarding both the therapeutic properties and potential side effects of cannabis among outpatients in northeastern Thailand, particularly those residing near the Thai–Cambodian border. Despite the legal liberalization of cannabis for medical and limited recreational use, over two-thirds of respondents answered “don’t know” to most knowledge-based items, including those related to both conventional and alternative medicine. The findings indicate that prior cannabis use, occupation as a village health volunteer, or even higher levels of education do not significantly improve health literacy concerning cannabis. These knowledge gaps are further exacerbated by legal ambiguities, cultural taboos, and fragmented regulatory communication. Consequently, the results underscore an urgent need for targeted public education campaigns, clearer legal frameworks, and accessible community-based health communication to promote safe, informed, and lawful cannabis use—especially in regions vulnerable to misinformation and cross-border trade challenges.

## Data Availability

No datasets were generated or analysed during the current study.
